# Supplementary figures and images for: The dual-specificity kinase DYRK1A interacts with the Hepatitis B virus genome and regulates the production of viral RNA
Source: PLoS One. 2024 Oct 15;19(10):e0311655. doi: 10.1371/journal.pone.0311655 (PMC11478819; doi:10.1371/journal.pone.0311655)

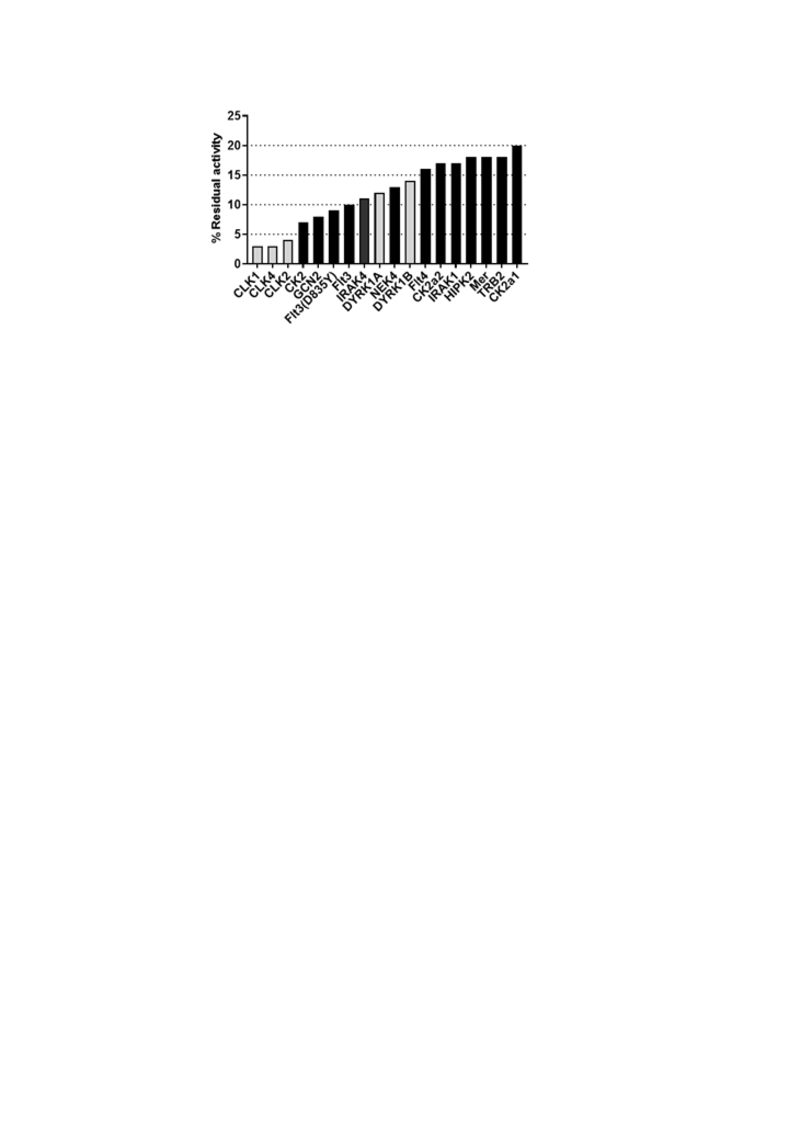

Supplement: S1 Fig — Only the kinases inhibited by 1C8 with a residual activity<20% are shown. Grey bars indicate the CLK and DYRK kinases targeted by the compound. (TIF) [file pone.0311655.s001.tif]

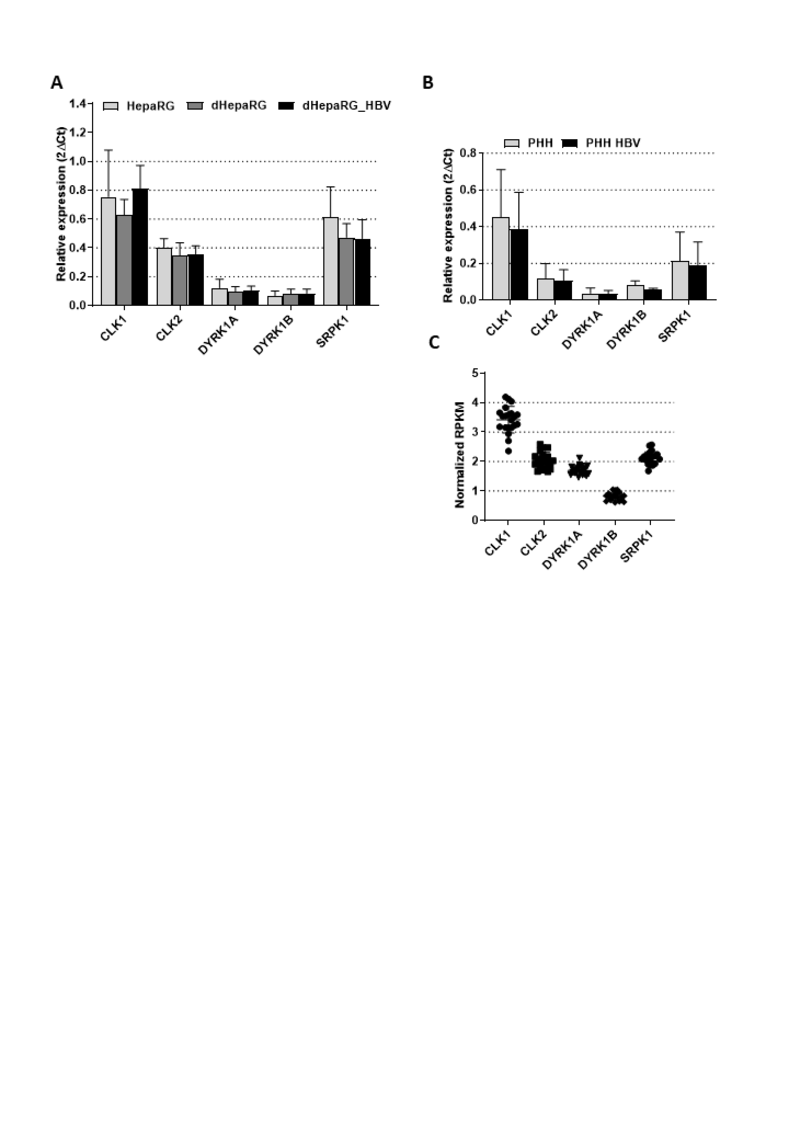

Supplement: S2 Fig — RNA levels of CMGC kinases indicated measured by RT-qPCR. Relative RNA levels are expressed by the 2ΔCt value calculated using the PRNP mRNA as control in HepaRG cells (undifferentiated or differentiated, (A) or PHHs (B), HBV-infected or mock-infected. PHHs used were purified from two different donors, each analyzed in triplicate. (C). CLK1, CLK2, DYRK1A, DYRK1B and SRPK1 mRNA expression in human liver using RNA-Seq data retrieved from Yoo et al. [75] expressed as Reads Per Kilobase Million (RPKM). (TIF) [file pone.0311655.s002.tif]

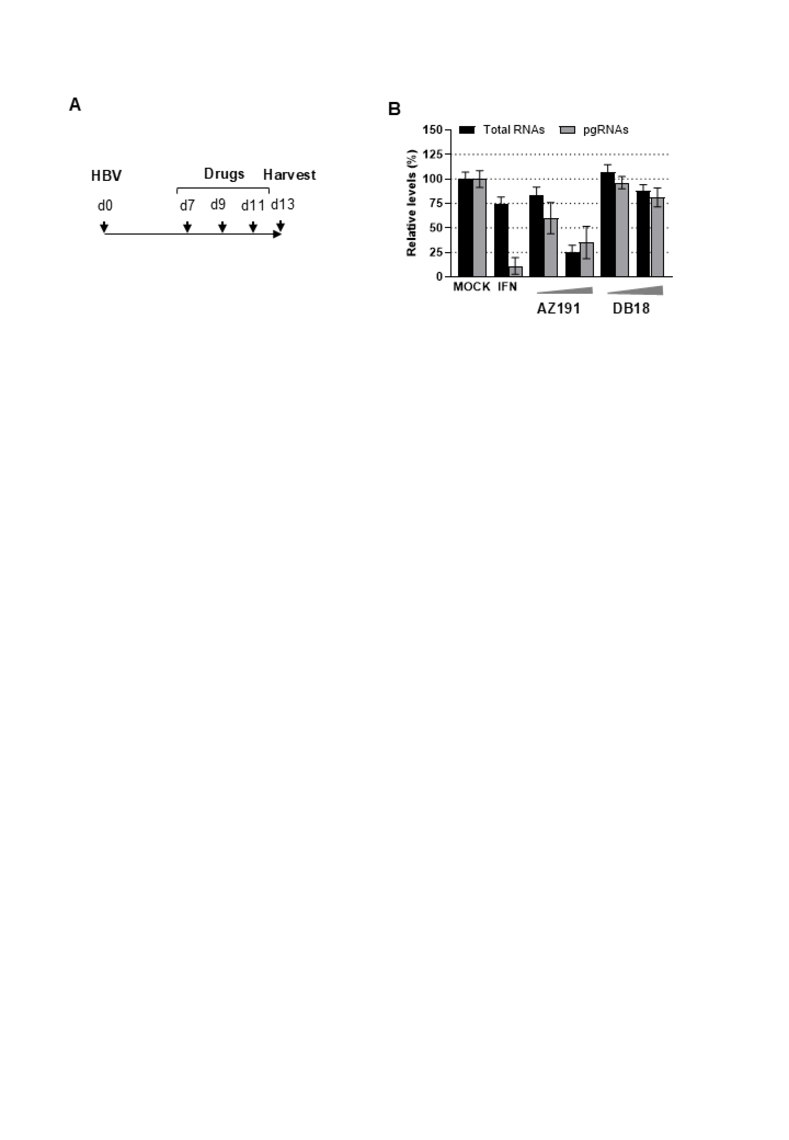

Supplement: S3 Fig — (A) Experimental outline. (B) PHH were infected and total viral RNAs and pgRNA were quantified by RTqPCR after treatment. IFN: Interferon-α (500 IU/mL). Doses of AZ191 and DB18 were 10 μM and 20 μM. Results are expressed as the mean +/- SD of two independent experiments, each performed in triplicate. (TIF) [file pone.0311655.s003.tif]

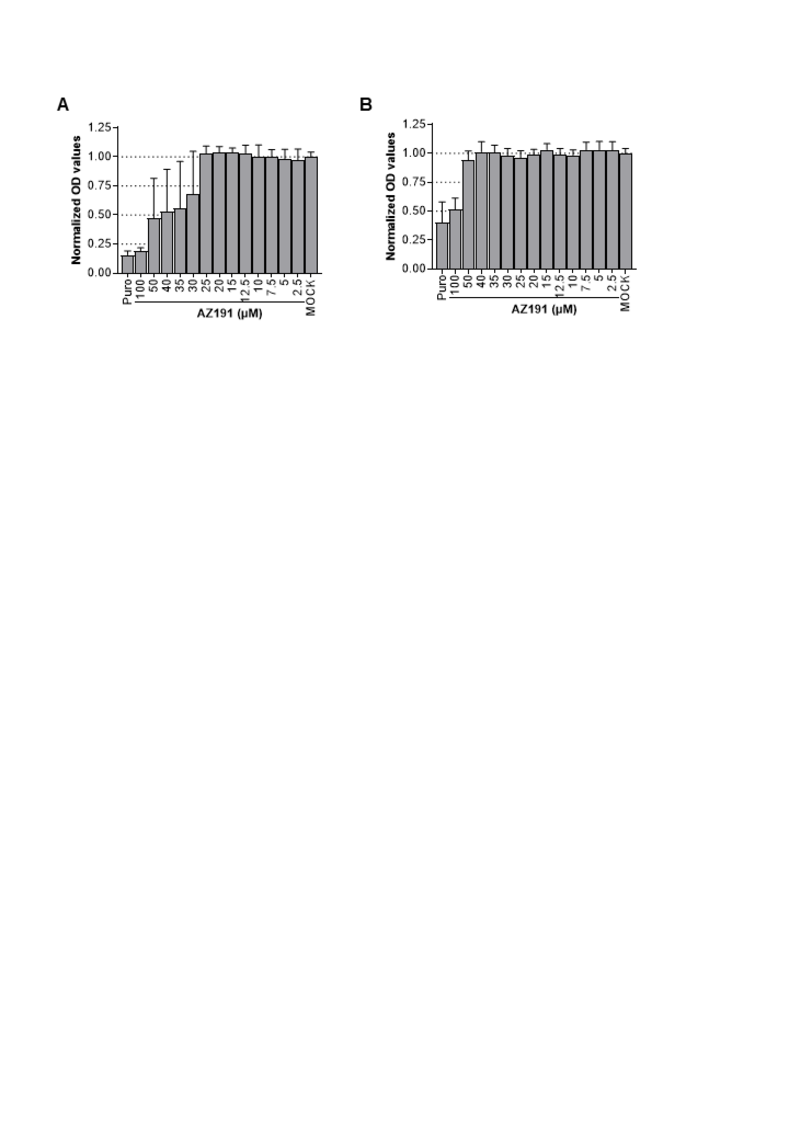

Supplement: S4 Fig — Cells infected and treated with various concentrations of AZ191, as indicated in Fig 2A, were analyzed for signs of toxicity using either a neutral red or a sulforhodamine B assay. Puromycin (Puro) was used as a positive control at 10 μM. Results are expressed as the mean +/- SD of 3 independent experiments, each performed in triplicate. (TIF) [file pone.0311655.s004.tif]

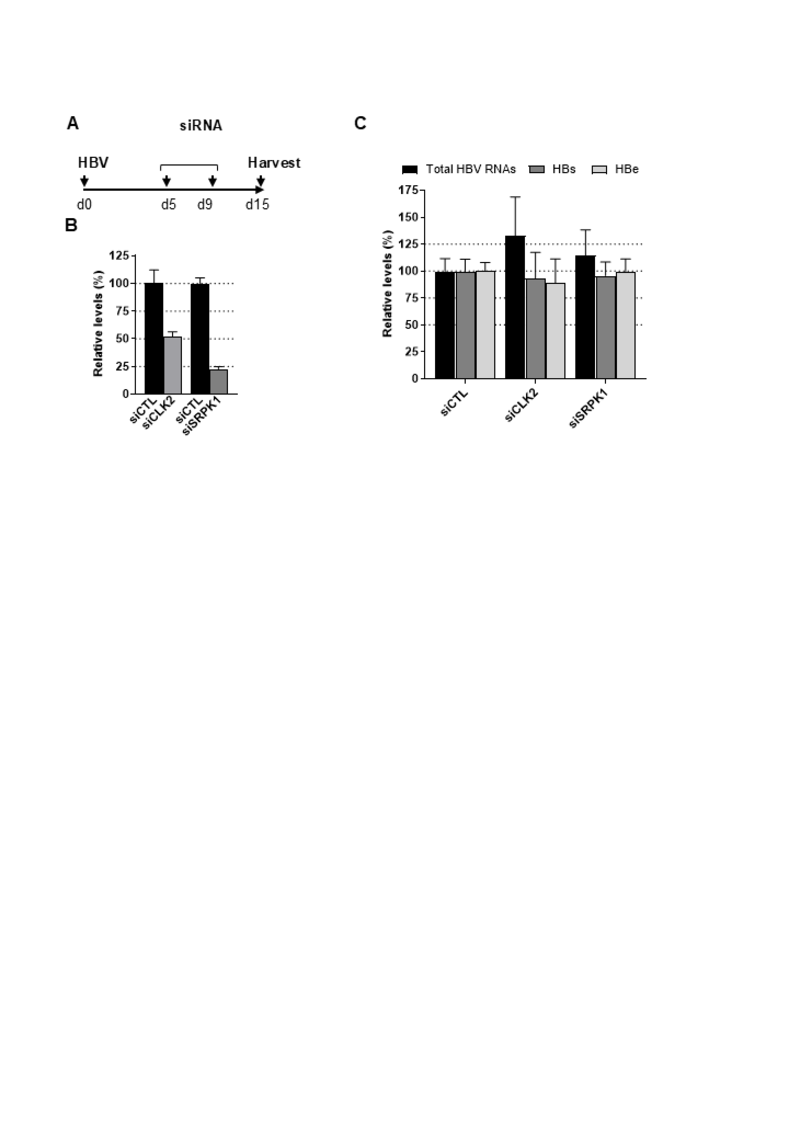

Supplement: S5 Fig — (A) Experimental outline. (B) KD efficiency of the siRNA treatments measured by RT-qPCR at 15-dpt. Results are shown relative to siCTL. (C) Effect of the kinases KD on the production HBV intracellular RNAs and secreted HBs/HBe antigens, expressed as relative to siCTL (mean +/- SD, n>5, each performed in triplicate). (TIF) [file pone.0311655.s005.tif]

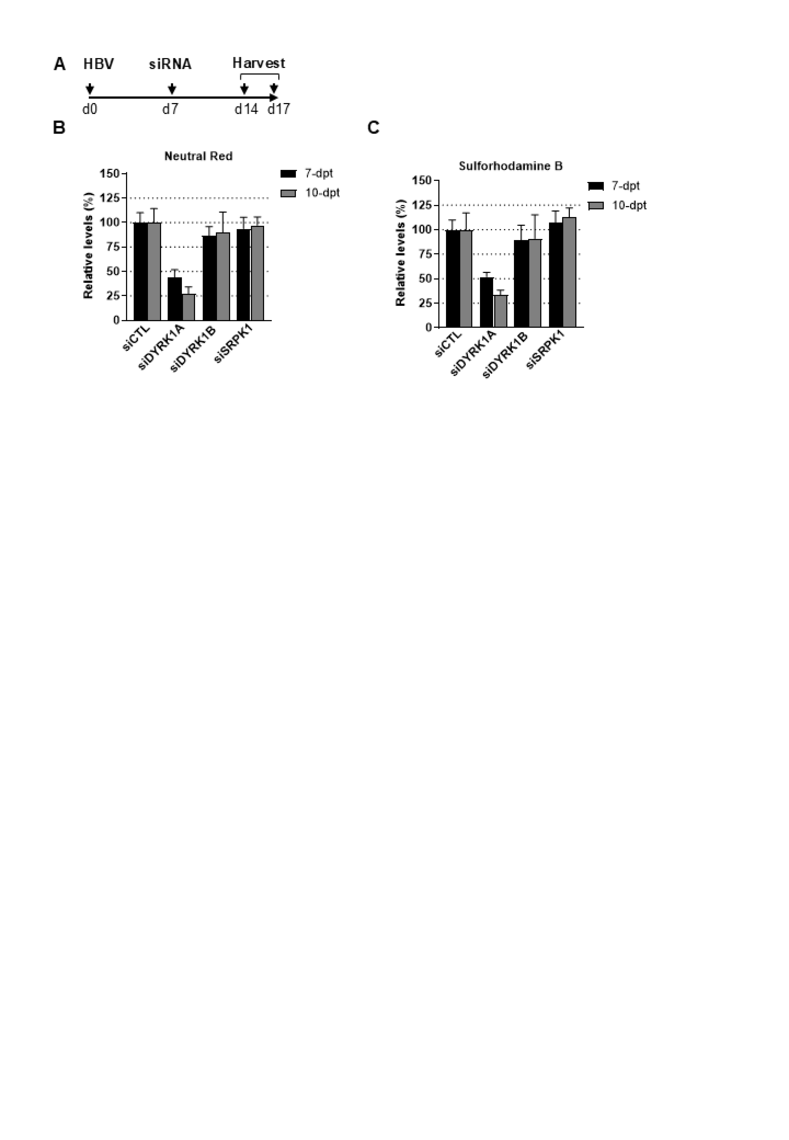

Supplement: S6 Fig — (A) Experimental outline. HBV-infected cells were transfected once with siRNA and then harvested at 7-, and 10-dpt. (B) and (C) Toxicity assays in siRNA transfected dHepaRG cells. Infected and transfected cells were analyzed for signs of toxicity using either a neutral red or a sulforhodamine B assay. Puromycin (Puro) was used as positive control at 10 μM. In all cases, results are shown relative to siCTL (mean +/- SD, n = 2). (TIF) [file pone.0311655.s006.tif]

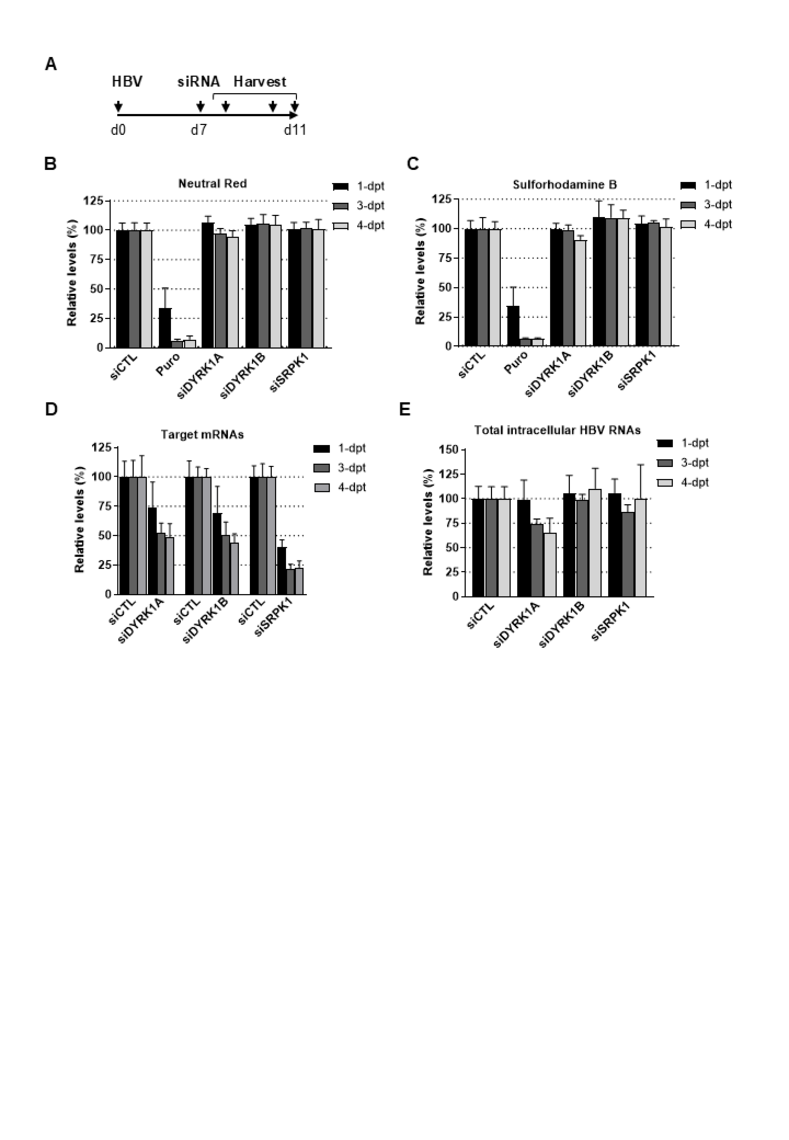

Supplement: S7 Fig — (A) Experimental outline. HBV-infected cells were transfected once with siRNA and then harvested at 1-, 2-, and 4-dpt. (B) and (C) Toxicity assays in siRNA transfected dHepaRG cells. Infected and transfected cells were analyzed for signs of toxicity using either a neutral red or a sulforhodamine B assay. Puromycin (Puro) was used as positive control at 10 μM. (D) KD efficiency of the siRNA treatments measured by RT-qPCR at the indicated time points. Results are shown relative to siCTL (mean +/- SD, n = 2–3). (E) Effect of the KD for the kinases indicated on HBV RNA production. Intracellular RNA extracted at each time point was analyzed by RT-qPCR to quantify HBV total RNAs. In all cases, results are shown relative to siCTL (mean +/- SD, n = 2). (TIF) [file pone.0311655.s007.tif]

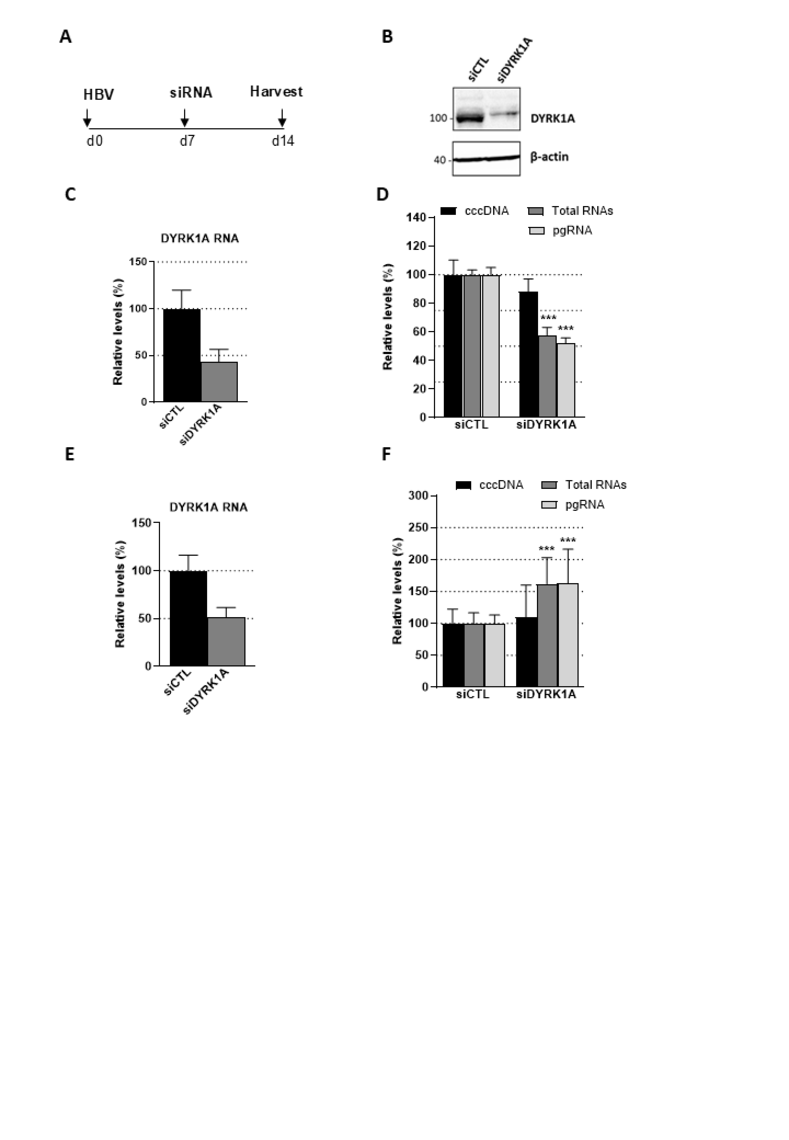

Supplement: S8 Fig — (A) Experimental outline. PHH were infected with HBV and 7 days later transfected with siRNA targeting DYRK1A or with a control siRNA (siCTL). (B) Representative Western blot analysis of DYRK1A KD levels. (C) and (D) DYRK1A and HBV RNA analysis of a set of 3 independent experiments, each performed in triplicate, in which a decrease of HBV RNAs was observed. (E) and (F) represent another set of 4 experiments in which HBV RNAs increased upon DYRK1A KD. Results are expressed as the mean +/- SD. Statistical analysis is from comparisons between siDYRK1A and control siRNA for each of the measurements. (TIF) [file pone.0311655.s008.tif]

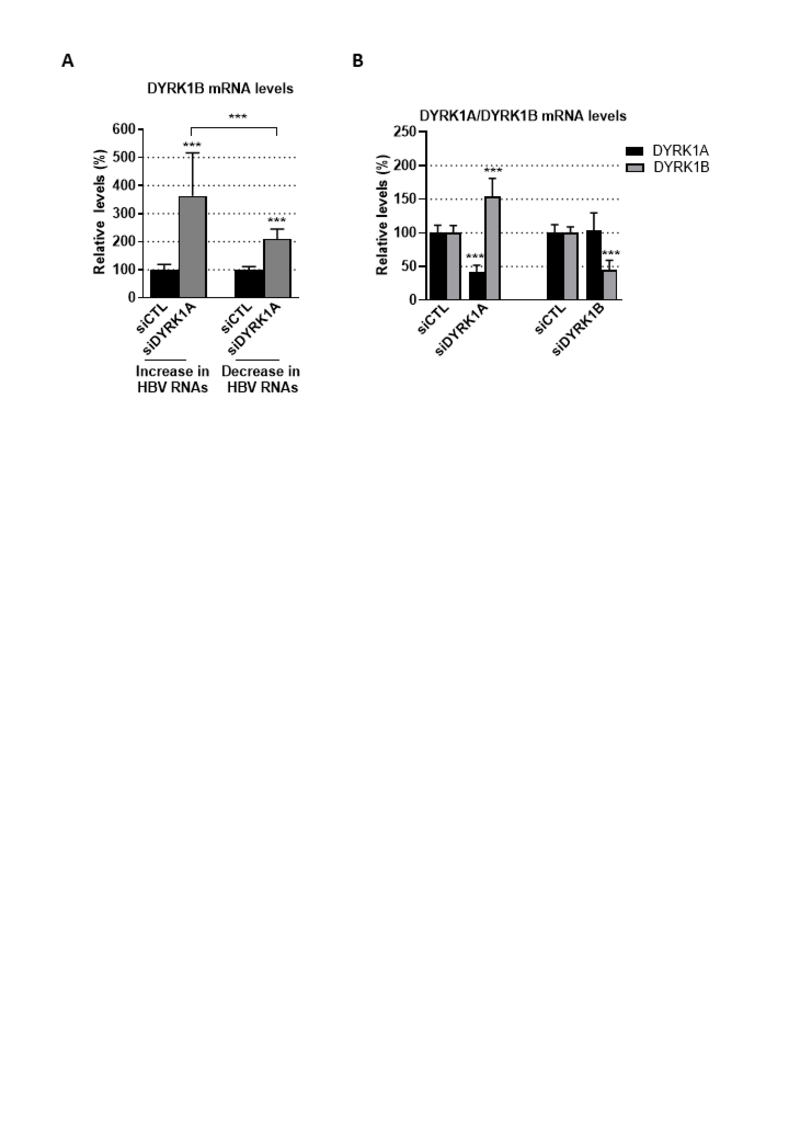

Supplement: S9 Fig — (A) mRNA levels were quantified in HBV-infected PHHs transfected with siRNA targeting DYRK1A as shown in S7A Fig. Results were clustered according to their effect on HBV RNA levels (see S7D and S7F Fig). (B) Analysis of DYRK1A/DYRK1B compensatory mRNAs variations in HBV-infected dHepaRG cells transfected with siRNA targeting either of the kinases. Statistical analysis is from comparisons between siRNA targeting one of the kinases and control siRNA for DYRK1A or DYRK1B mRNA levels. (TIF) [file pone.0311655.s009.tif]

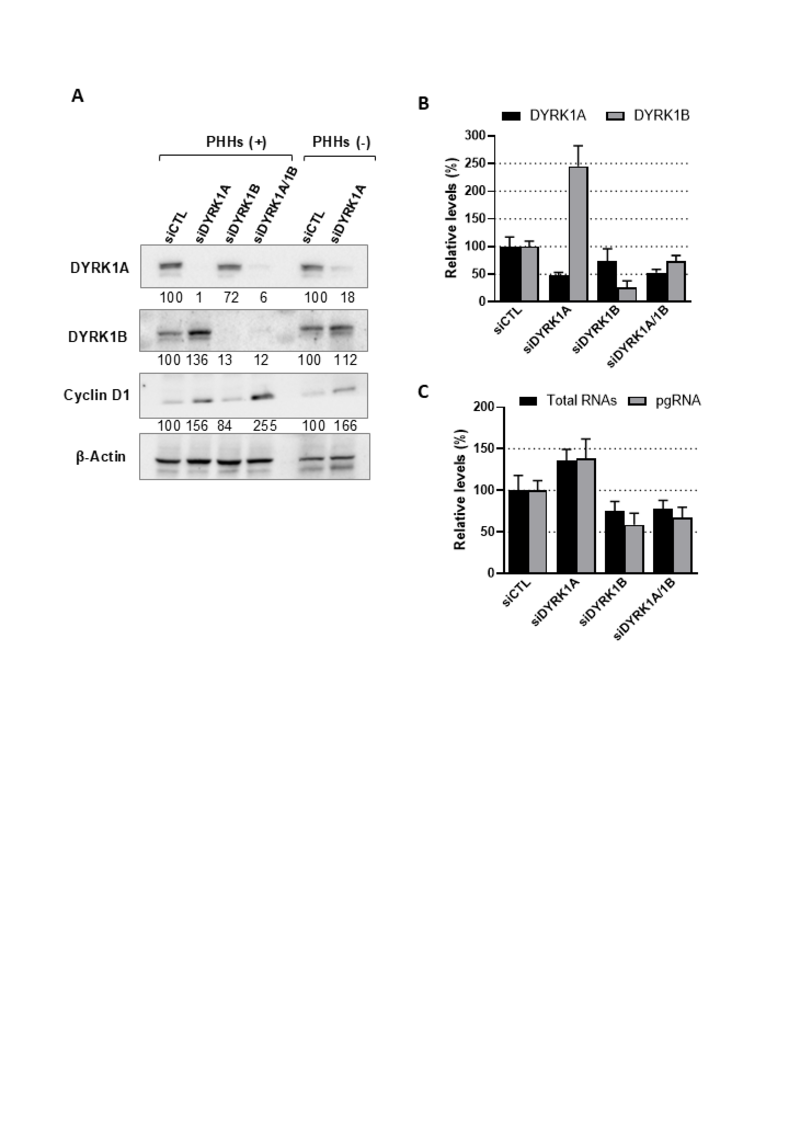

Supplement: S10 Fig — (A) DYRK1A and DYRK1B protein levels in HBV-infected PHHs with KD for each kinase. Cells were infected with HBV and then transfected with indicated siRNA. Western blot analysis was performed at 7-dpt. Cyclin D1 levels were analyzed as a marker of functional DYRK1 KD, since its accumulation has been shown to depend on both DYRK1A and DYRK1B [58, 76]. PHHs (+) and (-) refers to experiments in which an increase (+) or a decrease (-) of HBV RNAs levels was observed following DYRK1A KD (see S7 Fig). The bands were quantified using ImageJ (numbers relative to control siRNA, below the blots). (B) and (C) Effect of single DYRK1B or double DYRK1A/1B KD in HBV-infected PHHs. Cells were infected and transfected as indicated in S7A Fig. DYRK1A, DYRK1B (B), and HBV (C) RNAs were quantified at 7-dpt. Results are normalized versus control siRNA and expressed as the mean +/- SD of 2 independent experiments, each performed in triplicate. (TIF) [file pone.0311655.s010.tif]

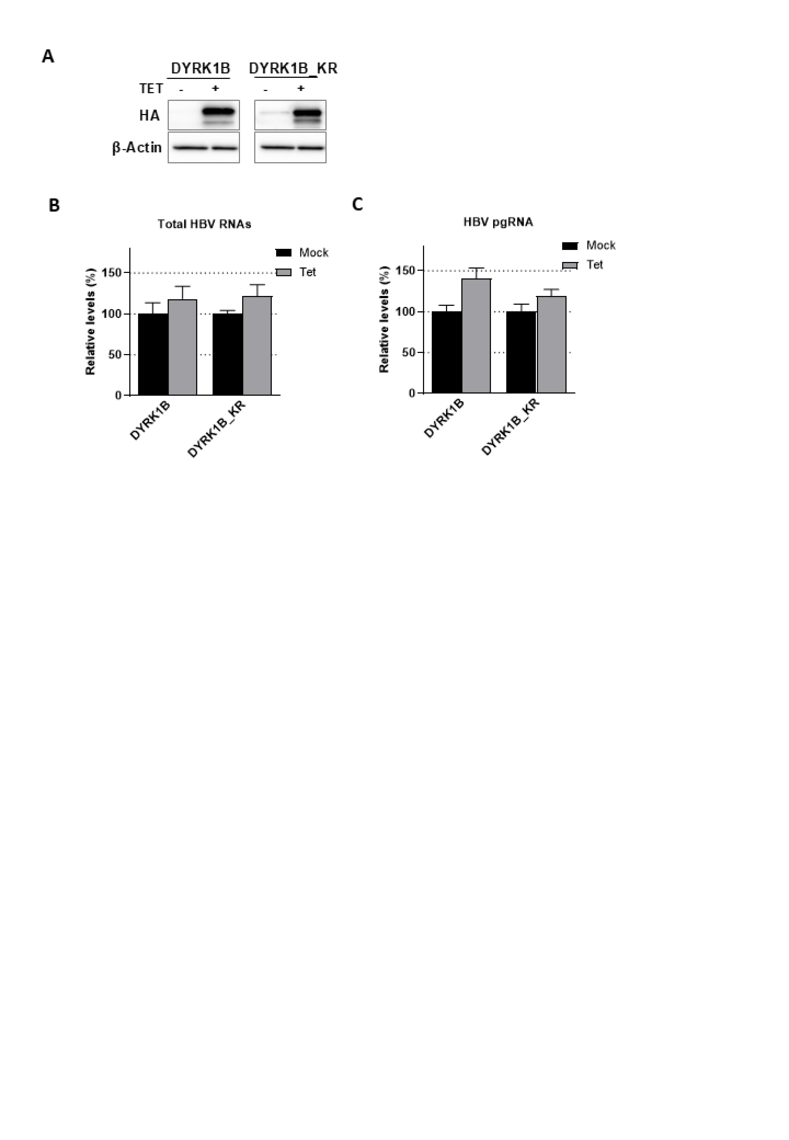

Supplement: S11 Fig — (A) dHepaRG cells engineered to express either HA-DYRK1A or HA-DYRK1A_KR under the control of a tetracyclin (Tet)-inducible promoter were infected with HBV and then treated with Tet, Tet plus 1C8, or mock treated. (B) to (E). Quantification of HBV viral parameters. Results are normalized to the mock situation and expressed as the mean +/- SD, of 2 independent experiments, each performed in triplicate. (TIF) [file pone.0311655.s011.tif]

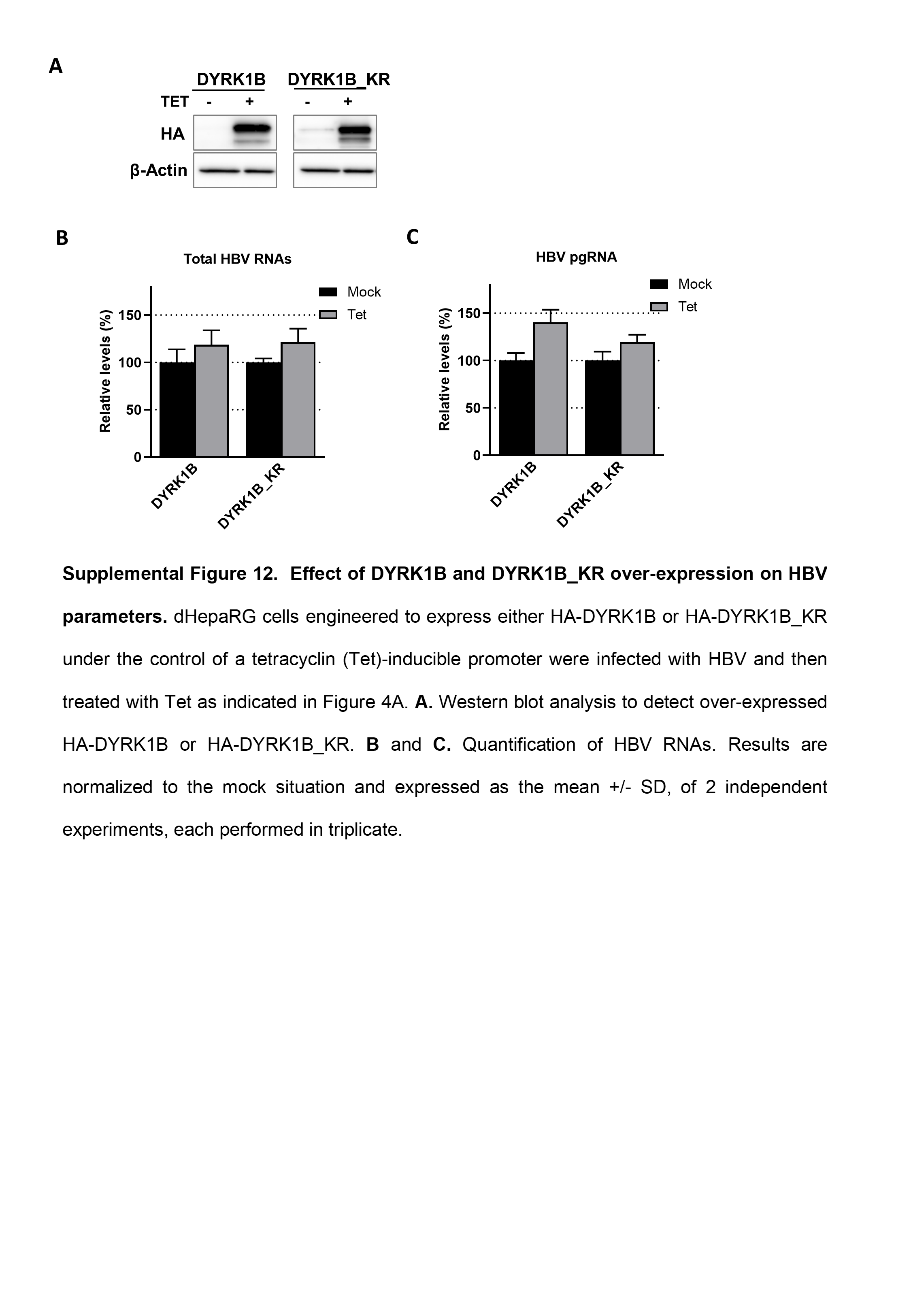

Supplement: S12 Fig — dHepaRG cells engineered to express either HA-DYRK1B or HA-DYRK1B_KR under the control of a tetracyclin (Tet)-inducible promoter were infected with HBV and then treated with Tet as indicated in Fig 4A. (A) Western blot analysis to detect over-expressed HA-DYRK1B or HA-DYRK1B_KR. (B) and (C) Quantification of HBV RNAs. Results are normalized to the mock situation and expressed as the mean +/- SD, of 2 independent experiments, each performed in triplicate. (TIF) [file pone.0311655.s012.tif]
