# Supplementary material for: The dual-specificity kinase DYRK1A interacts with the Hepatitis B virus genome and regulates the production of viral RNA
Source: PLoS One. 2024 Oct 15;19(10):e0311655. doi: 10.1371/journal.pone.0311655 (PMC11478819; doi:10.1371/journal.pone.0311655)
Supplement: S1 Raw images — (PDF) [file pone.0311655.s013.pdf]

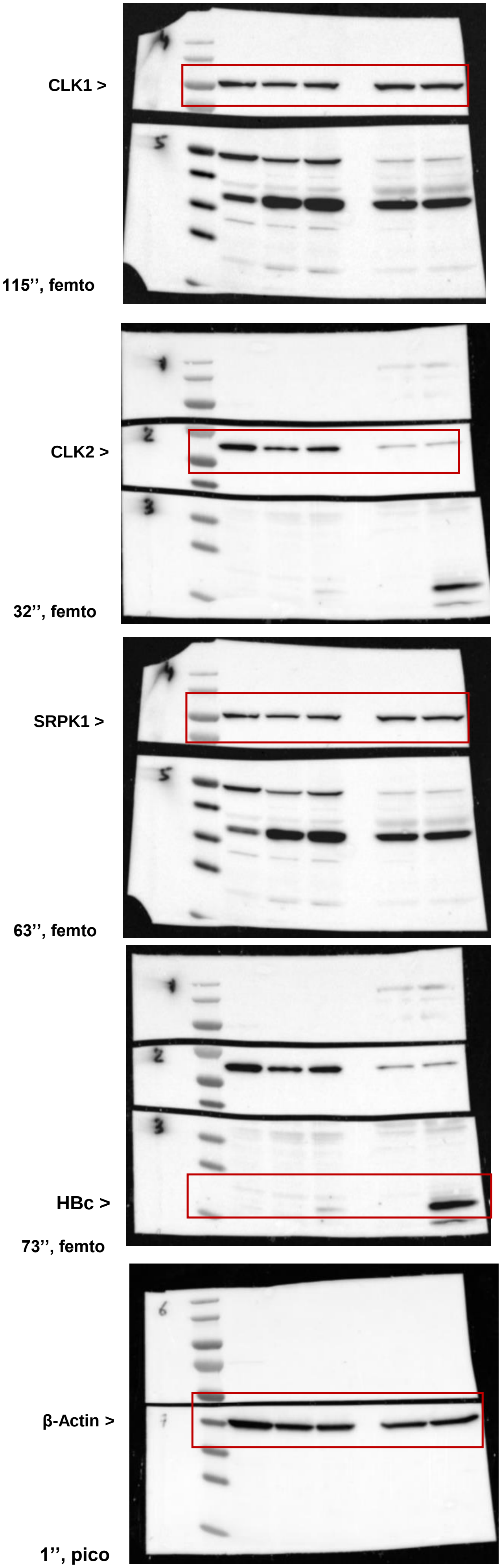

Figure 1 (left panel)

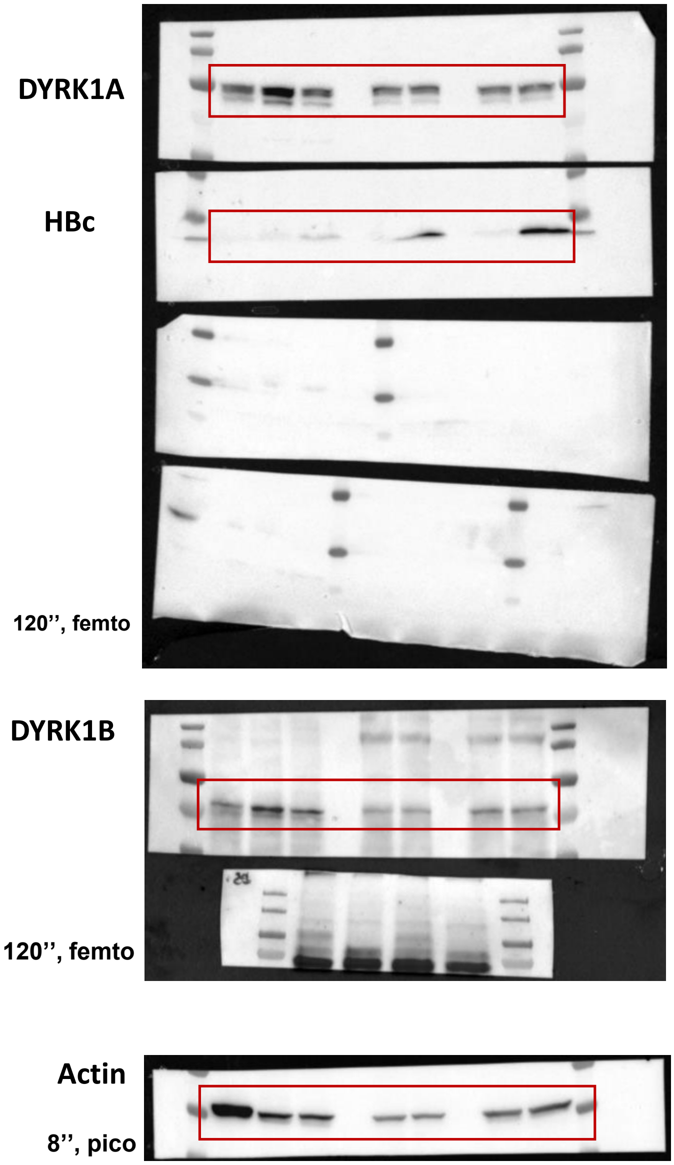

Figure 1 (right panel)

DYRK1A

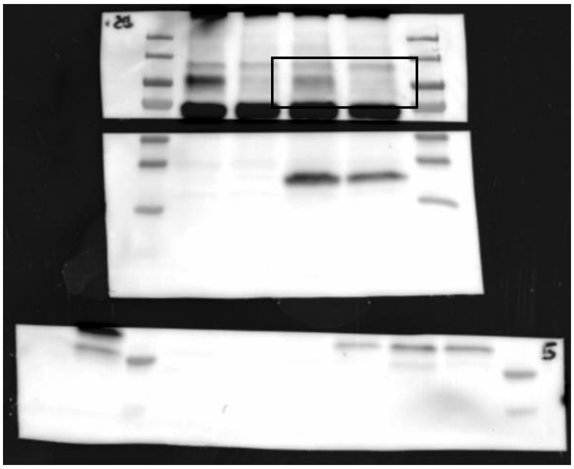

15'', femto

Actin

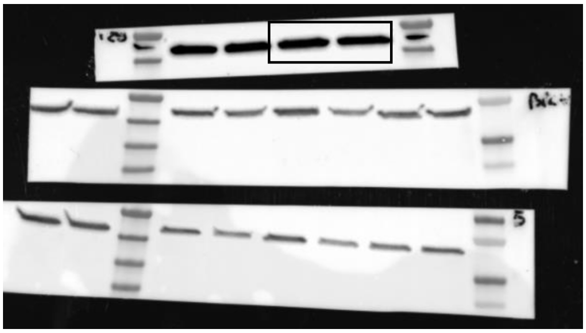

4'', pico

Figure 3

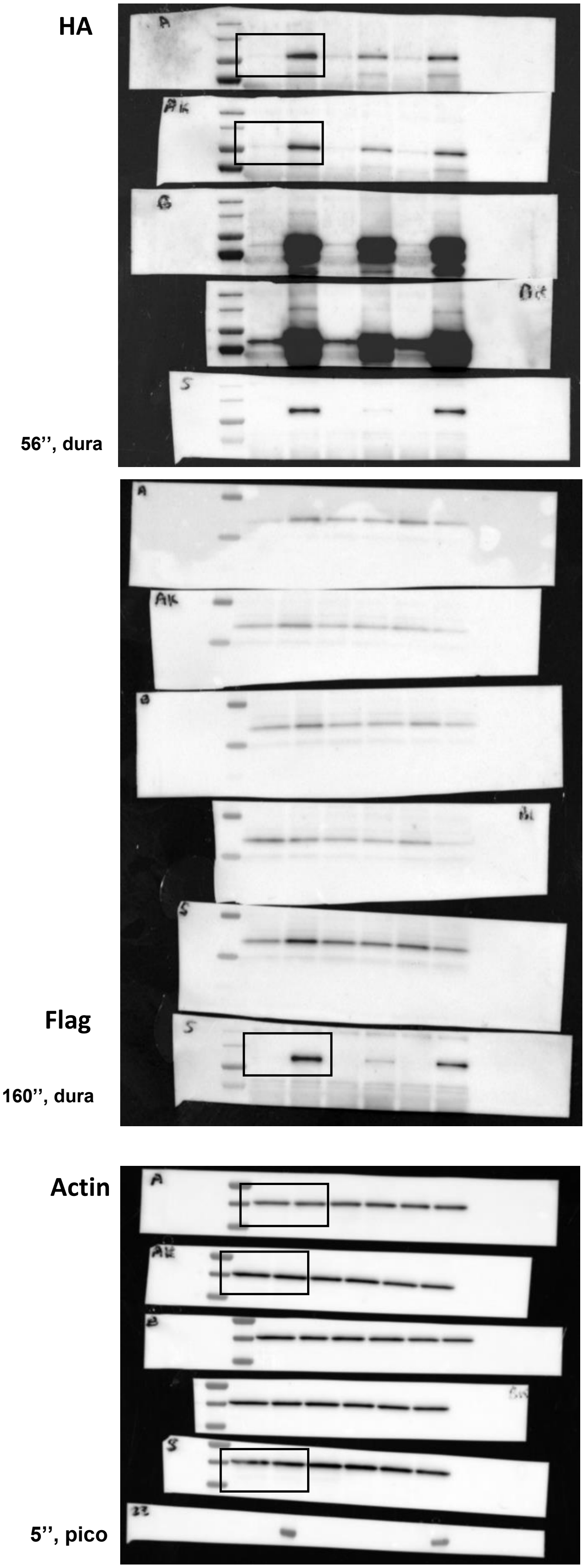

Figure 4

DYRK1A

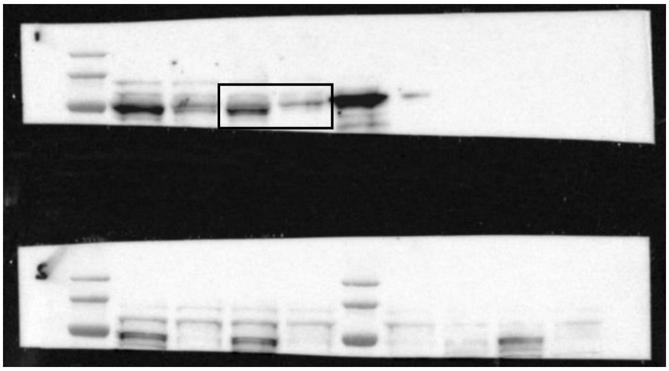

90’’, femto

Actin

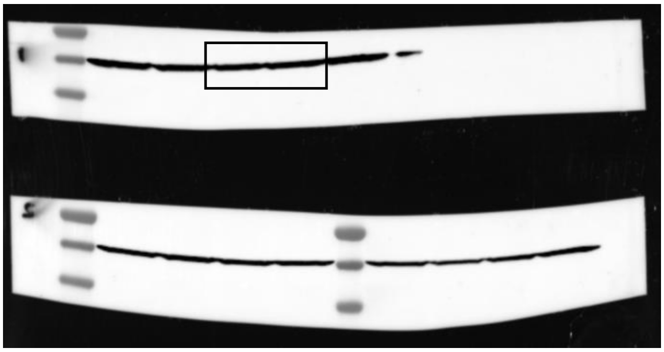

10’’, pico

Supplemental Figure 8B

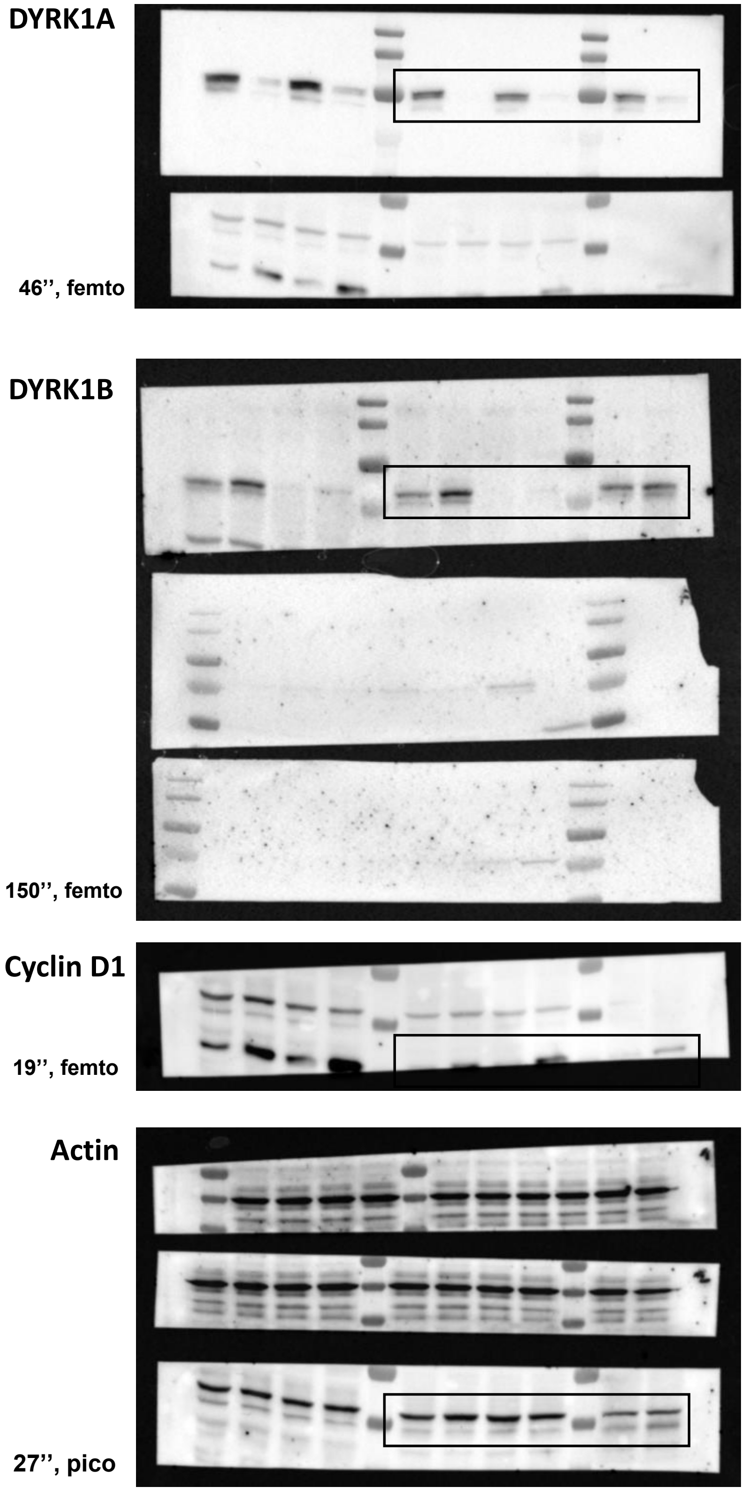

Supplemental Figure 10A

HA

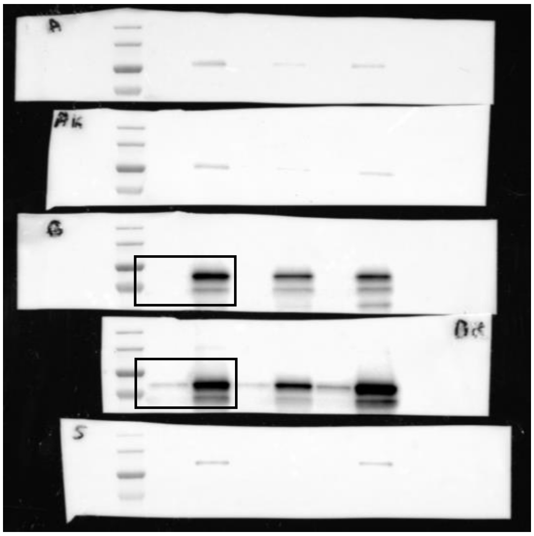

10'', dura

Actin

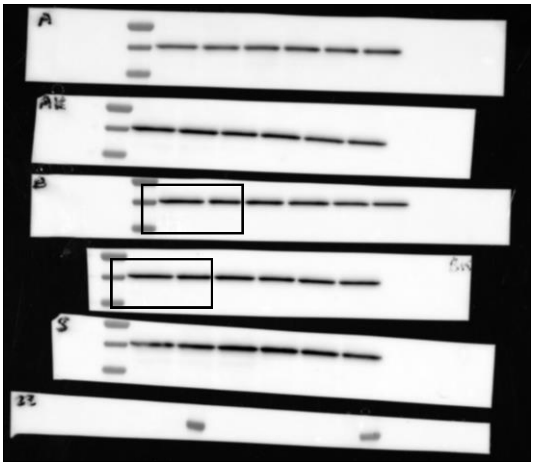

5'', pico

Supplemental Figure 12A
